# Doppler ultrasound protocol for patients with hidradenitis suppurativa^⋆^

**DOI:** 10.1016/j.abd.2023.10.003

**Published:** 2024-06-08

**Authors:** Ariany Tomaz de Aquino Saran Denofre, Carolina Meloni Stecca, Juliana Yumi Massuda Serrano, Thais Helena Buffo, Rachel Polo Dertkigil, Renata Ferreira Magalhães

**Affiliations:** a Discipline of Dermatology, Medical Sciences College, Universidade Estadual de Campinas, Campinas, SP, Brazil; b Discipline of Radiology, Medical Sciences College, Universidade Estadual de Campinas, Campinas, SP, Brazil

**Keywords:** Clinical protocols, Ultrasonography, Ultrasonography, Doppler

## Abstract

**Background:**

Hidradenitis suppurativa (HS) is a chronic inflammatory disease that leads to the formation of nodules, abscesses and fistulas, with the formation of scars and fibrosis, causing significant impairment in patient quality of life. The diagnosis is clinical, using scores to classify the severity of the condition; currently the most recommended classification is the International Hidradenitis Suppurativa Severity Scoring System (IHS4). Doppler ultrasound has been used to complement the clinical evaluation of patients with HS. It is possible to observe subclinical lesions that change the staging, the severity of the case, and its treatment, either clinical or surgical. Correct treatment is essential to minimize the consequences of this disease for the patient.

**Objective:**

To establish an outpatient protocol for the use of Doppler ultrasound in the care of patients with HS.

**Methods:**

A narrative review of the literature was carried out on the use of Doppler ultrasound in patients with hidradenitis suppurativa; a referring protocol and technique orientations for imaging assessment in HS were created.

**Results:**

Recommendation to perform ultrasound evaluation of symptomatic areas eight weeks after using antibiotics and four, 12, and 24 weeks after starting immunobiologicals; apply SOS-HS ultrasound severity classification.

**Study limitations:**

The review did not cover all literature on ultrasound and HS; no systematic review was carried out, but rather a narrative one.

**Conclusions:**

The correct assessment of patients staging must be carried out using dermatological ultrasound to avoid progression to scars and fibrosis, which compromise patients quality of life.

## Introduction

Hidradenitis suppurativa (HS) is a chronic, immune-mediated inflammatory disease of the hair follicle that causes the formation of nodules, abscesses and fistulas, mainly in the axillary, inguinal and anogenital regions, with the formation of bridging scars and fibrosis.^1^, ^2^, ^3^, ^4^ It is a recurrent and debilitating disease, that presents after puberty, leading to significant quality of life impairment by causing pain, pruritus, odor, and local burning sensation. It is the dermatological disease with the greatest psychosocial impact and highest Dermatology Life Quality Index (DLQI).^1^, ^5^, ^6^

To guide decisions regarding the treatment of HS, the clinical classification of subtypes is relevant, in addition to determining severity.^2^, ^4^ The patient approach involves both the management of worsening factors and treatment with medications and surgical procedures to remove fistulas and scars that act as recurrent inflammation sites. Thus, greater staging accuracy potentially impacts the therapeutic decision regarding the implementation of appropriate treatment proportional to the severity.^5^

Doppler ultrasound with a high-frequency transducer (above 15 MHz) has been used to characterize the type of lesion and its depth with greater precision,^2^, ^5^, ^7^, ^8^, ^9^ since the clinical palpation of lesions may not be accurate when differentiating nodules, abscesses and fistulas, especially when there is intense edema. This is essential for the evaluation and management of patients with HS.^8^, ^10^, ^11^

Compared to isolated clinical assessment, the use of ultrasound classifies a significant percentage of patients into more advanced stages, changing the therapeutic planning.^2^, ^8^, ^11^, ^12^ Its use changed the treatment in 82% of the assessed patients; in particular, medical treatment was changed to surgical in 24% of the cases.^11^

The main ultrasound findings in hidradenitis suppurativa are dilation of hair follicles, dermal changes, pseudocysts, fluid collections and fistulas. It has been proposed that the presence of three or more of these findings establishes the diagnosis of HS ‒ SOS-HS three-point score ‒ Sonographic Scoring of Hidradenitis Suppurativa.^11^ More recently it was also defined that there are five lesions observed in HS in the US: pseudocysts, collections, fistulas, fistulas and hair fragments (called hair tracts).^13^ Doppler ultrasound can also be used to identify subclinical fistulas and characterize inflammatory activity; it was established that there is an important correlation between the symptoms described by patients and ultrasound findings.^6^, ^12^, ^14^ A study showed that 76% of the assessed patients had fluid collections, 71% had pseudocysts and 29% had fistulas, all of which were not diagnosed on clinical examination.^11^

It is also possible to monitor the response to treatment non-invasively through vascularization assessment using Doppler^10^ and carry out better pre-operative planning for lesions that require surgical treatment. Moreover, there was a high correlation between ultrasound and histopathological findings. There is a relationship between Doppler intensity and neutrophilic infiltration; and good correlation between fistula diameter in ultrasound and histopathological evaluations.^15^ Similarly to the Hurley score, the SOS-HS was created using subclinical lesions, and shows a better correspondence with patient severity.^14^ Thus, ultrasound has shown to be an essential tool for greater accuracy in assessing the severity and extent of HS lesions, potentially impacting therapeutic decisions, allowing early and effective treatment to prevent complications, and the need for more invasive treatment. Therefore, the implementation of a Doppler ultrasound evaluation protocol as part of patients physical examination is necessary.^9^

## Materials and methods

A narrative review of the literature on the use of ultrasound in hidradenitis suppurativa was carried out to scientifically support the protocol. The search for articles was carried out in the VHL, EMBASE, SCOPUS, Web Of Science, and PUBMED databases, with 301 articles being retrieved. Ninety-six articles were selected after excluding duplicates and reading the titles and abstracts.

To guarantee the quality of the data collected for the protocol, articles published in journals classified by Qualis Periodics of the Sucupira Platform as A1, A2, and B1 were chosen, and five, three and eight articles remained, respectively (16 in total). Two books published on the subject of dermatological ultrasound were also selected.

The extracted data were used to prepare the protocol, which contains recommendations based on the reviewed articles with the highest scientific level. Then, patients who had been attended since March 2021 at the Hidradenitis suppurativa outpatient clinic at the Dermatology outpatient clinic were recruited for ultrasound recording of the lesions and incorporation into the protocol.

## Results

### Protocol for the use of Doppler ultrasound in hidradenitis suppurativa

Why use ultrasound in hidradenitis suppurativa?

Ultrasound is an important tool for the early and accurate diagnosis of skin lesions, especially when they are small or not palpable.^10^ It also helps identify subclinical fistulas,^16^ which can affect disease staging.^8^, ^10^, ^13^, ^17^^,^^18^ Clinical scores alone may underestimate the degree of disease involvement. Additionally, Doppler ultrasound allows mapping of the extent and activity of skin lesions, facilitating the assessment of treatment effectiveness.^10^, ^13^, ^15^ Corticosteroid infiltration can be guided by ultrasound to improve precision, with better results.^19^, ^20^ In the case of fistulas, ultrasound helps to determine the depth, which influences the appropriate treatment, whether with immunobiologicals or surgical. The combined use of immunobiologicals and ultrasound before surgery is effective in preventing recurrence and accurately determining surgical margins. Additionally, ultrasound can be used to evaluate possible recurrences after surgery.^10^, ^21^

### Severity classification

The patient should be examined and the scores used to classify severity before performing the ultrasound. Then ultrasound severity scores should be applied. The easiest ultrasound score is the SOS HS (Table 1),^15^, ^18^, ^23^ which uses the number of ultrasonographic detected lesions (in a similar way to Hurley) to define stages I, II or III, with the latter being the most severe.

**Table 1:** Ultrasonographic score for the classification of hidradenitis suppurativa -SOS HS (Sonographic Scoring of Hidradenitis suppurativa).

**Stage**	
I	One fluid collection and changes in the dermis (anechoic or hypoechoic pseudocystic nodules, increase in hair follicles, changes in thickness and echogenicity of the dermis)
II	Two to four fluid collections or a fistulous tract with dermal changes, affecting up to two body segments (uni- or bilateral)
III	Five or more fluid collections or two or more fistulous tracts with dermal changes or involvement of three or more body segments (uni- or bilateral)

### Interval between exams

Ultrasound can be used to monitor response to treatment. It should be performed at the first consultation,^15^, ^18^ after eight weeks of antibiotic use,^15^ in weeks four, 12 and 24 when using immunobiologicals,^24^ 12 weeks after infiltration^19^, ^20^ and six months after surgery to evaluate postsurgical recurrence.^21^

### Technical aspects

Ultrasound must be configured with the focal point in the first 3 cm from the top of the screen and must be adjusted for dermatological ultrasound.^17^ The frequency, gain and image volume must be kept constant to prevent problems with reproducibility.^15^, ^23^

Dermatological ultrasound can use a frequency between 13.5 and 100 MHz, with 20-25 MHz examining the epidermis and dermis and 50-100 MHz restricted to the epidermis.^25^ The recommendation is to use several probes between 5‒22 MHz.^8^, ^10^, ^13^, ^14^, ^15^, ^22^, ^23^, ^24^, ^26^ It is always recommended to use a linear probe.^8^, ^14^, ^22^, ^23^^,^^27^

The examination should preferably be performed by the same investigator,^14^, ^15^, ^22^, ^23^ who should be trained in the ultrasound technique and in dermatology^13^, ^17^, ^21^, ^26^ or by dermatologists with at least 10 years experience in HS and two ultrasound operators who have completed training courses.^18^

### Performance

When performing Doppler ultrasound examinations in patients with HS, it is important to follow a sequence of steps to ensure the effectiveness of the procedure. First, it is necessary to perform the examination after the clinical assessment of the patient, taking into account the established criteria.^8^, ^18^ Additionally, it is essential to to explain the procedure to the patient and obtain information about lesion evolution.

Before starting the examination, it is recommended to apply an adequate amount of gel to the area to be examined or directly to the ultrasound probe.^14^, ^17^ Subsequently, visual inspection and palpation of the lesional and perilesional area are performed in a well-illuminated place. For a better observation, it is important to reduce the light in the room and position the lesion close to the operator and the probe.^17^

**Figure 1:**
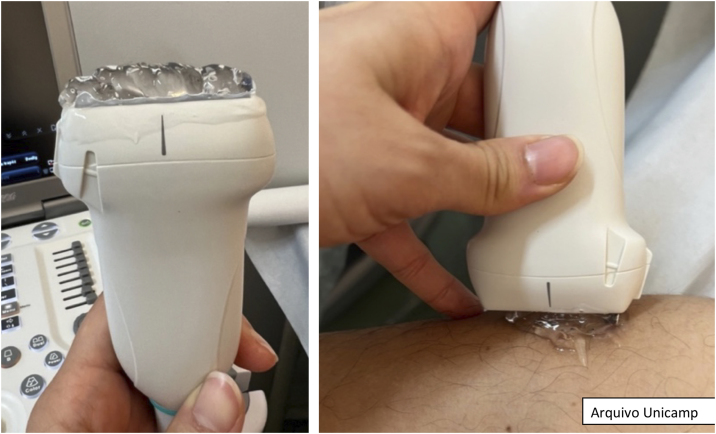
Amount of gel to be used and perpendicular positioning of the probe.

During the examination, the probe should be gently positioned perpendicular to the lesion, as shown in Fig. 1.^27^ It is important to apply little pressure and stabilize the hand with the help of the fifth finger. Image capture must be done in two perpendicular axes, covering both the lesional and perilesional areas.^8^, ^10^, ^14^, ^17^^,^^22^ If necessary, one can compare the images with others obtained from the contralateral area.

Moreover, Color Doppler analysis is recommended to evaluate the vasculature pattern of the lesional and perilesional area, as well as evaluate vessel caliber. If necessary, the spectral curve can be used to differentiate between veins and arteries and detect flow velocity.^17^

### Ultrasound assessment of lesions

During the evaluation of patients with HS, it is necessary to follow a sequence of steps for a complete and systematic analysis. First, it is important to evaluate all existing lesions,^8^, ^27^ in at least two different regions.^10^ Additionally, it is essential to always examine the axillary and inguinal regions, as well as the perineal and perianal areas.^8^, ^10^, ^18^ If it is impossible to do it that way, evaluate at least all symptomatic areas.^15^

For a standardized approach, it is recommended to use a specific sequence, starting from top to bottom and medial to lateral regions. During the examination, it is necessary to identify the type of lesion according to its characteristics and describe the dermal changes.^10^ It is also important to measure the size of the lesions and the maximum diameter of nodules, abscesses and fistulas. The precise location of the lesions must be described.^10^, ^15^, ^23^, ^24^^,^^27^

Numbers can be used to facilitate the identification and recording of lesions, for example, C1, C2 for collections, and F1, F2 for fistulas. If the patient has multiple fistulas, it is recommended to select the largest one for a more accurate evaluation.^21^, ^28^

**Figure 2:**
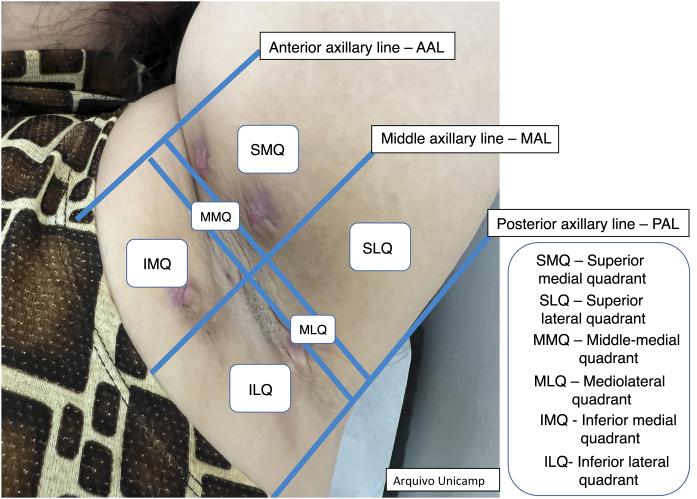
Axillary and intermammary region.

**Figure 3:**
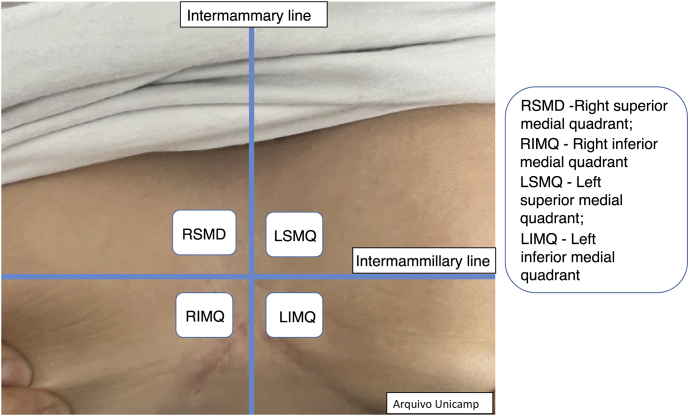
Intermammary region.

**Figure 4:**
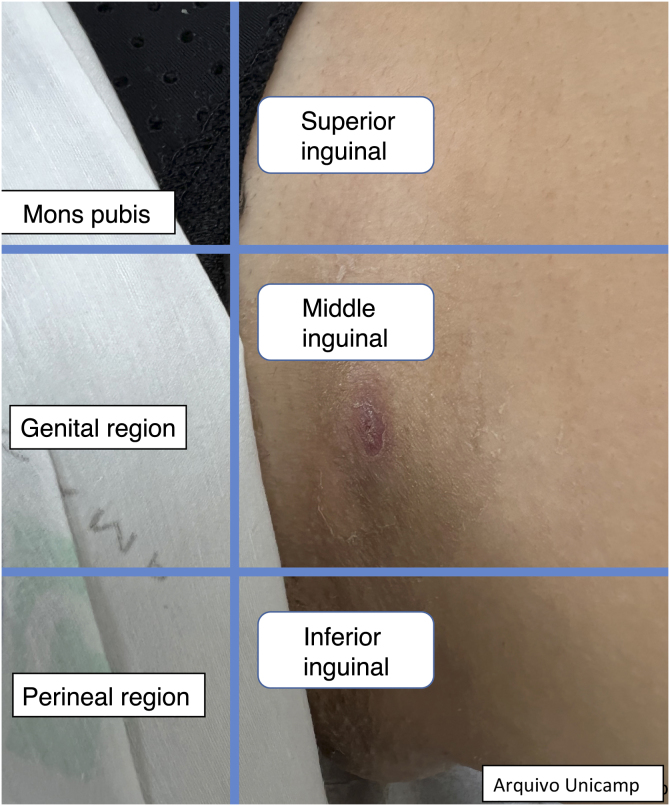
Inguinal region.

Moreover, describing the exact location of each lesion can be challenging due to the number of lesions. Therefore, the authors suggest the use of the quadrant classification of the UQHS anatomical areas – Ultrasound Quadrants in Hidradenitis Suppurativa (Figure 2, Figure 3, Figure 4) to assist in the description of the report. This classification was designed using anatomical references such as the anterior, middle and posterior axillary lines, to form the quadrants. It is also possible to incorporate the description of lesions in “hours” and outer, middle and inner sections (for each quadrant). For example, in Fig. 2, there is a lesion in the superior medial quadrant of the left axilla, at 12 o'clock, in the inner section. Thus, it is possible to locate the lesions more precisely.

### Classification of UQHS anatomical areas

Figure 2, Figure 3, Figure 4.

### Ultrasound structures

The ultrasound findings already described in the literature are:^11^, ^13^, ^16^

1. Dilatation of hair follicles.
2. Changes in dermal thickness and echogenicity (Fig. 5).

**Figure 5:**
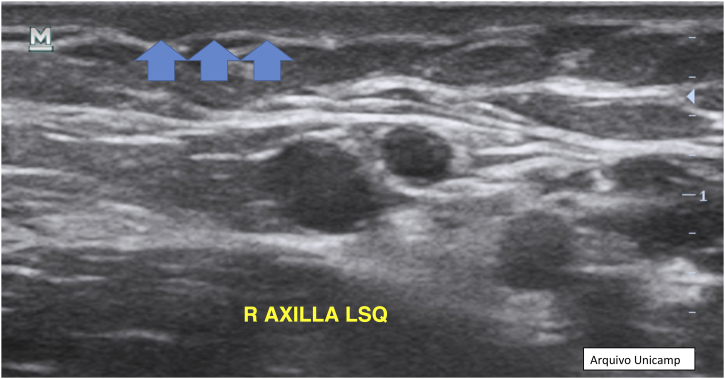
Changes in the thickness and echogenicity of the dermis.
3. Anechoic or hypoechoic dermal pseudocysts – round or oval hypoechoic and anechoic structures in the dermis and/or hypodermis measuring <1 cm, which may clinically correspond to nodules or abscesses (Fig. 6).

**Figure 6:**
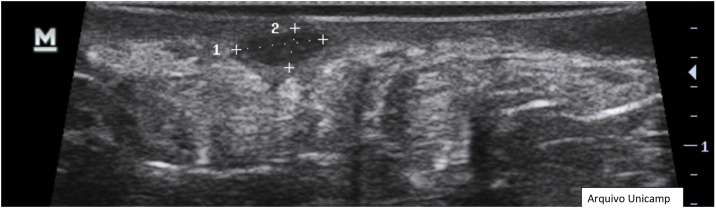
Pseudocyst.
4. Anechoic fluid collections with echoes (debris) – hypoechoic or anechoic dermal fluid and/or hypodermic sac-like structure connected to the base of the enlarged hair follicle, clinically corresponding to an abscess or fistula (Fig. 7).

**Figure 7:**
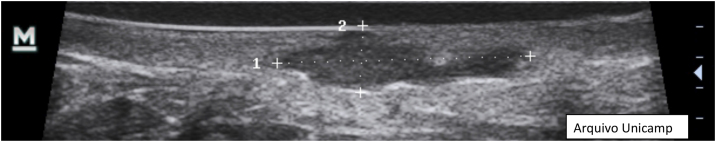
Fluid collections.
5. Hypoechoic fistula/fistulous tracts in the dermis and subcutaneous tissue – dermal or hypodermal hypoechoic or anechoic band-like structure connected to the base of an enlarged hair follicle (Fig. 8).

**Figure 8:**
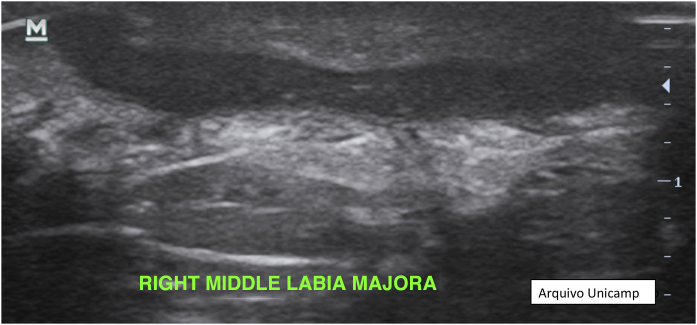
Fistulae.

**Figure 9:**
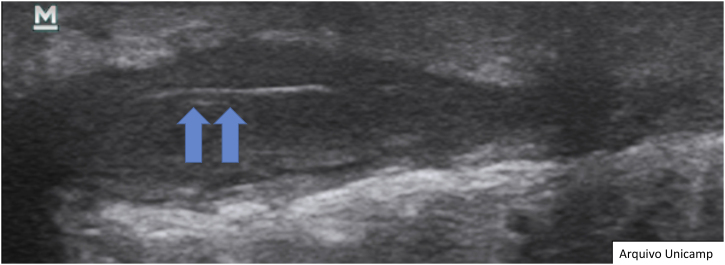
“Hair tracts”.

More recently, an international consensus of experts validated, in addition to the abovementioned lesions, “hair tracts”, or fragments of hair, with a trilamellar, bilaminar or monolaminar appearance and an hyperechoic linear pattern (Fig. 9).^9^, ^13^

### Complementary evaluations

**Figure 10:**
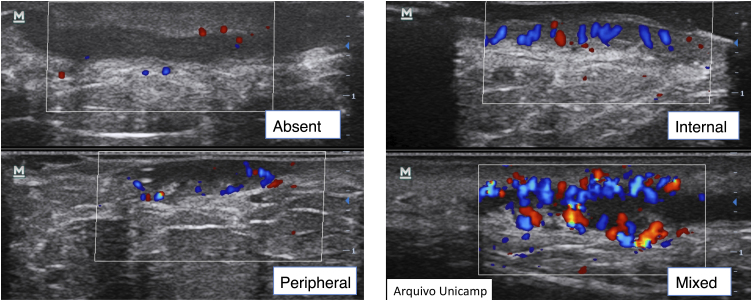
Type of vascularization on Doppler.

Vascularization evaluation with Doppler provides an objective assessment of the degree of inflammation and disease activity. It can be classified as absent, peripheral, internal or mixed (Fig. 10).^15^, ^22^, ^23^, ^24^

It is also possible to grade the amount of vascularization^23^ as high, if there are multiple signs, moderate if there are some signs of flow, minimal if there are a few points and absent. Nodules and abscesses have a peripheral vascularization pattern, simple fistulas, peripheral and mixed, and complex fistulas show a mixed pattern. Nodules show moderate to low amount of vascularization^20^ and abscesses and fistulas show a high to moderate degree of vascularization.^13^, ^23^ As the disease becomes chronic, the vascularization changes from peripheral to mixed.^23^

**Figure 11:**
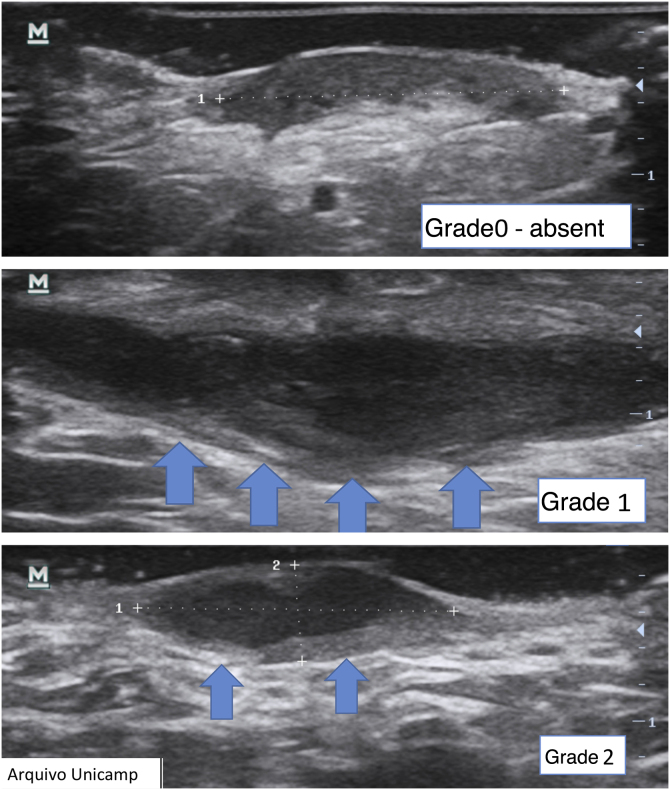
Grading of fibrosis.

Another important complementary assessment is the grading of fibrosis (Fig. 11),^16^ which may appear as absent (grade 0), as a thin peripheral hypoechoic band with a fibrillar pattern (grade 1) or as a thick, peripheral hypoechoic band with a fibrillar pattern that invades the fistula lumen and produces a hypoechoic halo that is transversely visible (grade 2).

A fistula with a high degree of fibrosis is chronic and requires surgical treatment.

**Figure 12:**
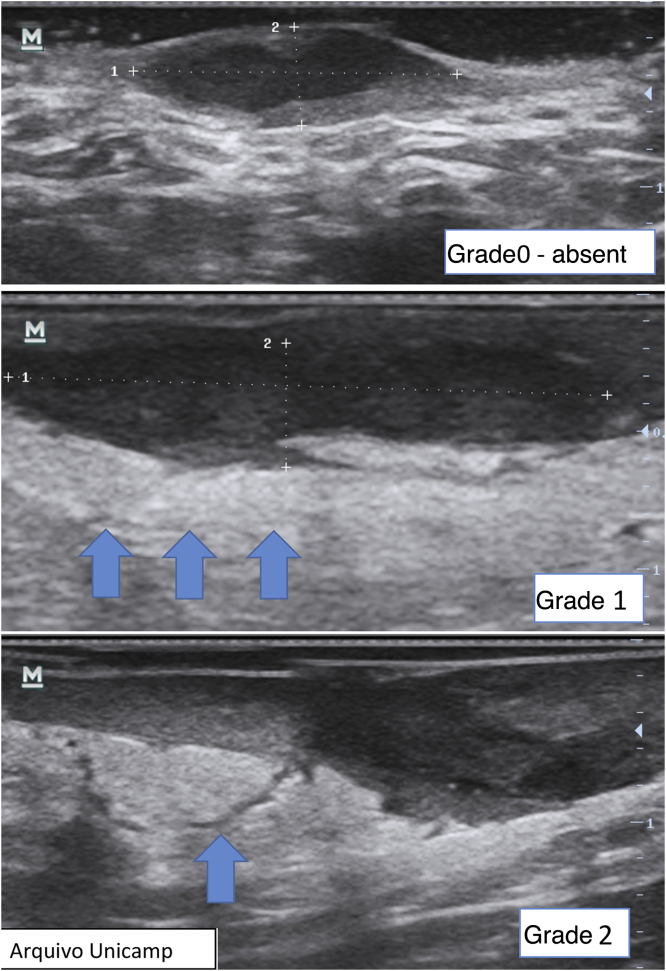
Grading of perilesional edema.

Finally, it is also possible to observe and grade perilesional edema, which directly correlates with inflammation and associated symptoms such as pain (Fig. 12).^22^, ^24^ It may be absent (grade 0), show hypodermic hyperechogenicity (grade 1) or hypodermic hyperechogenicity and anechoic fluid among the hypodermal adipose lobes (grade 2).

**Figure 13:**
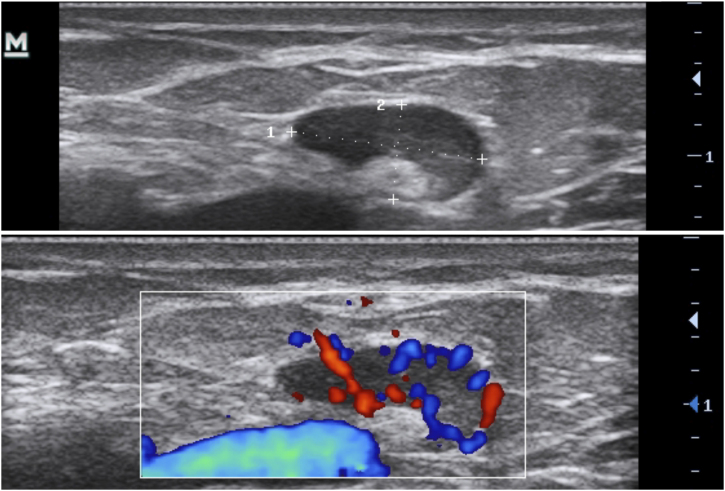
Reactive lymph node, without important changes.

Another possible finding are reactive lymph nodes that have an elliptical well defined shape, with hypoechoic cortical thickening, and an hyperechoic medulla in the central area (Fig. 13).^17^, ^23^, ^28^ Normally they are 1 cm in diameter and the Doppler assessment shows hilar vessels in the central area or at one of the borders.^10^, ^17^, ^25^

### Ultrasound during surgery

Determination of surgical margins intraoperatively through ultrasound examination can prevent recurrence. The technique used to determine ultrasound surgical margins involves first determining a 0.5 cm width clinical margin and then explore the area identified with ultrasound, transversely. Then, increase the area to be excised if subclinical lesions are present, repeating the process until there are no more lesions.^22^

### Recording of medical information^17^

The medical record should contain the type of lesion, anatomical location (Figs. 2‒4), diameter, vascularization status, description of relevant surrounding structures and inflammatory activity and degree of edema using Doppler.

### Device care^17^

As HS lesions may be draining fluids, it is recommended to use plastic covers for the probe, without significantly compromising the images obtained. After each patient examination, the probe should be cleaned with an alcohol solution.

### Limitations for the use of ultrasound

Despite the various advantages, available ultrasound devices do not allow the identification of lesions smaller than 0.1 mm, and do not show pigment or lesions exclusively intraepidermal,^8^, ^17^ with no real impact in the assessment of HS.

## Discussion

Dermatological ultrasound is important in the care of patients with HS because it allows the identification of the types of lesions at the time of the examination with greater accuracy.^14^ Comparisons between clinical and ultrasound indices^9^ demonstrated that clinical propaedeutics frequently underestimates patient severity, which impacts the therapeutic planning. Ultrasound also allows objectively assessing the response to treatment using Doppler,^23^ with planning of fistulae treatment according to their characteristics^5^ and more precise surgical planning with less recurrences.^22^

The patient with HS deals with acute exacerbations (“flares”) in addition to the sequelae of previous lesions. As patients take an average of 7.2 years^29^ to get a precise diagnosis, the multiple active and inactive lesions, in addition to fibrosis, make it difficult to accurately assess the lesions. The nodules, abscesses, and different types of fistulas require different therapeutic interventions. Superficial fistulas can be treated with deroofing, while deeper ones must be treated with conventional surgical excision.^5^

There is no previous protocol in the Brazilian literature providing guidelines on the use of ultrasound in HS. This protocol used the main evidence from the literature, covering all HS findings, with the aim of improving care for these patients. It is possible that future therapies will be guided according to ultrasound findings, with prediction of the onset and response to treatment according to ultrasound follow-up.^22^

## Conclusion

A complete evaluation of the anatomical area and degree of inflammation in a non-invasive, live and interactive way in patients with HS, is provided by dermatological ultrasound examination, in comparison with clinical examination. This protocol allows guiding its use in the care of patients with HS, improving follow-up and treatment of these patients.

## Financial support

None declared.

## Authors' contributions

Ariany Tomaz de Aquino Saran Denofre: Design and planning of the study; data collection, or analysis and interpretation of data; drafting and editing of the manuscript; collection, analysis and interpretation of data; intellectual participation in the propaedeutic and/or therapeutic conduct of the studied cases; critical review of the literature.

Carolina Meloni Stecca: Collection, analysis and interpretation of data; effective participation in research orientation; intellectual participation in the propaedeutic and/or therapeutic conduct of the studied cases.

Juliana Yumi Massuda Serrano: Collection, analysis and interpretation of data; effective participation in research orientation; intellectual participation in the propaedeutic and/or therapeutic conduct of the studied cases.

Thais Helena Buffo: Collection, analysis and interpretation of data; effective participation in research orientation; intellectual participation in the propaedeutic and/or therapeutic conduct of the studied cases.

Rachel Polo Dertkigil: Collection, analysis and interpretation of data; effective participation in research orientation; intellectual participation in the propaedeutic and/or therapeutic conduct of the studied cases.

Renata Ferreira Magalhães: Design and planning of the study; critical review of important intellectual content; effective participation in research orientation; intellectual participation in the propaedeutic and/or therapeutic conduct of the studied cases; approval of the final version of the manuscript.

## Conflicts of interest

None declared.
